# Outstretching challenges for rehabilitation of a mucormycotic case-a digitally designed patient-specific implant approach in the recent era

**DOI:** 10.1186/s12903-024-05099-4

**Published:** 2024-11-15

**Authors:** Ankita Pathak, Mithilesh Dhamande, Bhushan Mundada, Anjali Bhoyar, Seema Sathe, Smruti Gujjelwar, Shubham Tawade, Aashish Gupta, Prasanna Sonar

**Affiliations:** 1 Prosthodontics and Crown and Bridges, Sharad Pawar Dental College and Hospital, Datta Meghe Institute of Higher Education and Research, Sawangi (M), Wardha, India; 2 Oral and Maxillofacial Surgery, Sharad Pawar Dental College and Hospital, Datta Meghe Institute of Higher Education and Research, Sawangi (M), Wardha, India; 3 Oral Medicine and Radiology, Sharad Pawar Dental College and Hospital, Datta Meghe Institute of Higher Education and Research, Sawangi (M), Wardha, India

**Keywords:** COVID-19, Patient-specific implants, Mucormycosis, 3D printing technology, Post-mucormycosis rehabilitation, Prosthetic dentistry

## Abstract

**Background:**

Reconstruction of maxillofacial defects is challenging due to the region’s complex anatomy. During the COVID-19 era, many patients lost their maxilla and chewing efficiency as a result of Mucormycosis. In such cases, custom-designed implants offer a graftless solution for seemingly hopeless situations. This case report aims at miraculous dental transformation utilizing the Patient Specific Implant (PSI) approach.

**Case Presentation:**

A 64-year-old male patient presented to the Prosthodontics Department with the chief complaint of missing teeth. He had been struggling with chewing for two years due to a post-mucormycotic maxillary jaw. A CT scan was obtained to evaluate and investigate the affected site. After a comprehensive diagnosis, the treatment of choice was a patient-specific implant decided using 3D printing technology. Reconstructing maxillofacial defects poses significant challenges due to the region’s intricate anatomy, as well as its aesthetic and functional implications. The use of pre-formed alloplastic implants and autogenous grafts often leads to complications such as resorption, infection, and displacement. However, recent technological advances have made it possible to fabricate customized patient-specific implants (PSIs) through computer modeling, offering new opportunities for reconstructive surgery.

**Conclusion:**

This case report demonstrates the dental management of post-mucormycotic patients with specially designed implants, customized according to the availability and anatomy of the bone in the entire head region. The absence of complications during follow-up, conducted at 15, 30, 45, and 90 days, and subsequently monthly for two years, highlights the success of this approach. Evaluation parameters included infection, soft tissue recovery, wound separation, masticatory efficiency, stability of the prosthesis, and aesthetic outcomes. The positive outcomes observed at follow-up appointments emphasize the viability and effectiveness of patient-specific implants in addressing maxillary defects caused by post-mucormycosis.

**Supplementary Information:**

The online version contains supplementary material available at 10.1186/s12903-024-05099-4.

## Background

During the second wave of the COVID-19 pandemic, India faced a notable increase in mucormycosis cases. Nonetheless, rehabilitating such patients poses challenges, especially considering factors like age and defect severity. This case report aims to introduce an innovative digital methodology for designing patient-specific implants (PSIs) and assess their efficacy. Mucormycosis, a rapidly spreading fungal infection primarily affecting elderly individuals with compromised immunity, was previously a relatively unfamiliar condition until the emergence of the second wave of the COVID-19 pandemic in 2019. Between April and July 2021, India saw a notable increase in incidence, with over 45,000 cases recorded; the Rhinocerebral form accounted for around 77.6% of cases [1].

Complicated and extensive maxillofacial defects often lead to facial asymmetry, coupled with functional and aesthetic abnormalities, significantly impacting a patient’s psychological well-being [2]. Moreover, addressing these defects presents challenges owing to their distinctiveness. The coupling of three-dimensional (3D) printing with computer-aided design-computer-aided manufacturing (CAD-CAM) has revolutionized the field of reconstruction since the early 1940s, as it has made it easier to fabricate customized implants (PSIs) for individual patients [3].

Computed tomography (CT) scans are used to precisely determine the anatomy of the defects, allowing for the replication of the patient’s defect site into a 3D printed model, which offers exact replication of details and improved adaptation at the affected region [4, 5]. Such an approach aids in mitigating the drawbacks associated with autogenous grafts, such as the risk of infections, graft resorption, suboptimal cosmetic outcomes, and donor site morbidity [6]. The current case report addressed surgical and prosthetic considerations through real-time communication to fabricate PSIs.

To stop the disease’s spread, later stages of the illness often require a whole or partial maxillectomy. After removing the necrotic bone, the primary closure method involves using the buccal and palatal mucosa. Due to their age and the extent of the impairment, these individuals pose major rehabilitation challenges. Following that, a plethora of surgical and prosthetic complications occur, including a lack of maxillary bones, involvement of the zygomatic bone or pterygoid plates, adhesion of the sinus and nasal mucosa with fibrosed palatal mucosa, reduction of the stress-bearing area, loss of lip support, inadequate vertical guidance, and mandibular overclosure. In these situations, returning to normal function with the least amount of morbidity and guaranteeing long-term sustainability should be the main goals of rehabilitation. Conventional maxillary reconstruction techniques usually entail augmentation using free bone grafts to place endosseous implants. Nevertheless, there are several drawbacks to this method, including the requirement for further surgical operations, varying rates of graft resorption, extended rehabilitation times, and secondary donor site morbidity. Although quad zygoma is still a possibility in certain situations, it is not feasible for individuals whose zygomatic bone has been lost due to the removal of necrotic bone [7].

Advancements in oral and maxillofacial surgery, including cone beam computed tomography (CBCT), intraoral scanners, and CAD/CAM software, have significantly enhanced precision in designing and manufacturing patient-specific implants (PSIs). The direct metal laser sintering technique (DMLS) offers new opportunities for fabricating custom-made PSIs tailored to each patient’s anatomical needs [8]. These digital advancements allow for revisiting older techniques like subperiosteal implants, which, although previously abandoned, can now be utilized in a noninvasive manner with improved outcomes [9]. Such implants can be a boon for rehabilitating post-maxillectomy cases, such as those following mucormycosis-related maxillectomy [10, 11].

To construct a titanium patient-specific implant (PSI) for a patient who underwent hemimaxillectomy due to a post-COVID mucormycosis infection, this case study effectively utilized three-dimensional (3D) technology. The discussion focused on the issues surrounding occlusal function, aesthetics, and facial asymmetry that arise after excision following mucormycosis. After undergoing treatment for mucormycosis and having a subtotal maxillectomy defect repaired with an implant customized for the patient, the patient was discharged.

With teeth replaced immediately, positive clinical results were observed, including improved function, enhanced facial symmetry, and better psychological well-being. This case study demonstrates how the use of PSIs for complex maxillofacial anomalies has been revolutionized by combining 3D printing and computer-aided design/computer-aided manufacturing (CAD-CAM) technologies.

### Case report

**Fig. 1:**
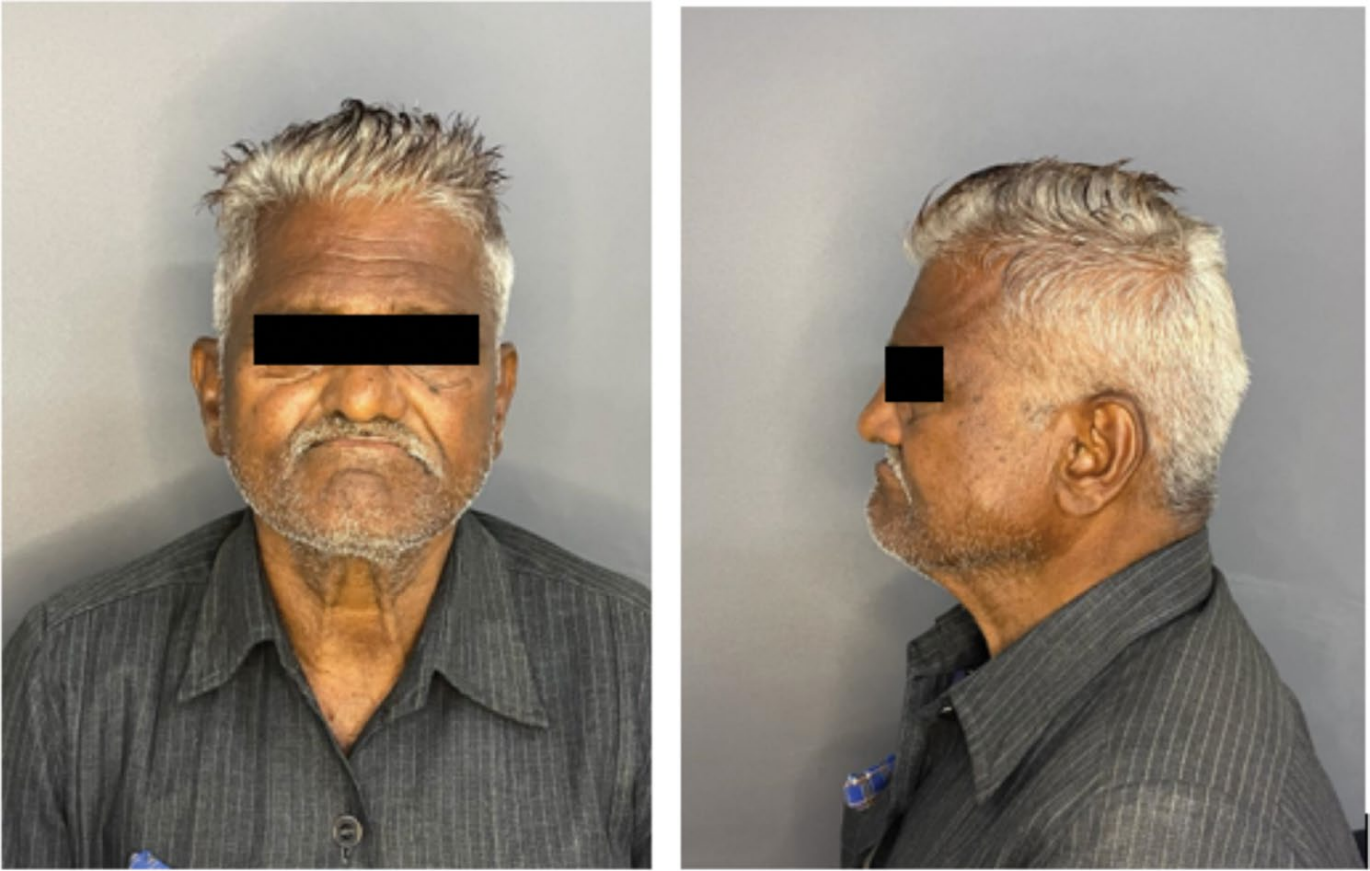
Front profile and lateral profile

A mucormycotic-afflicted male patient, 67 years old, who had undergone subtotal maxillectomy with primary closure and was symptom-free for more than a year post-surgery, was reported to the Department of Prosthodontics. The history of the presenting illness includes the patient being diagnosed with a condition known as mucormycosis a year ago, which infected the patient’s upper jaw. The patient tested positive for SARS-CoV (Coronavirus disease) and was treated for the same for 6 months—resection of the maxilla was performed during surgery due to the mucormycosis infection. No significant habit history was noted for the patient. The patient’s chief complaint was difficulty in mastication and speech. The individual’s face exhibits a square shape, with symmetric facial features. His facial profile was straight, and the temporomandibular joint (TMJ) displayed smooth and synchronous movement on both sides. Mandibular movement was bilaterally symmetrical. Lip competency was observed as competent, with lips slightly inverted. The middle one-third of the face presents a slightly concave profile, with a noticeable reduction in vertical height (Fig. 1).

**Fig. 2:**
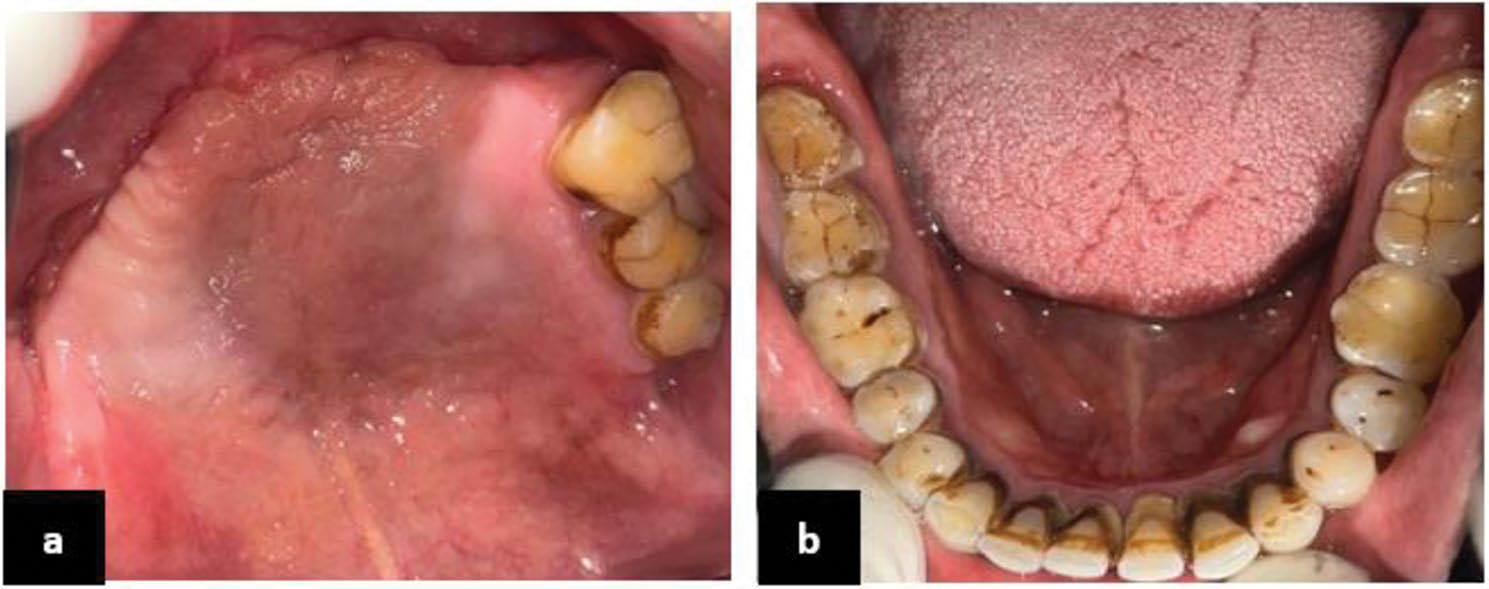
**a** Upper arch, **b**: Lower Arch

**Fig. 3:**
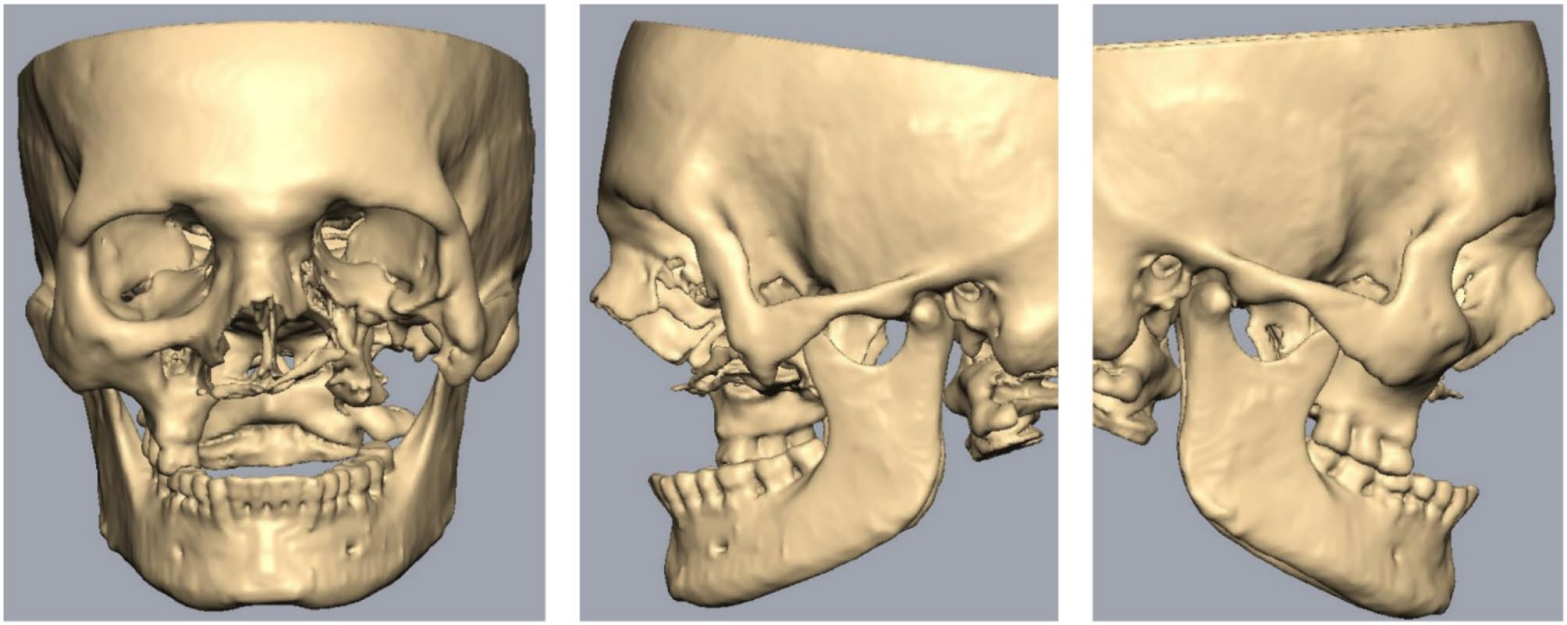
CT scan

An intraoral and radiographic examination with Computed Tomography (CT) was performed. The CT Scan was conducted to interpret and analyze the severity and extent of the lesion (Figs. 2 and 3).

After the CT scan was evaluated, it was observed that the patient had lost all bone in the maxilla and had insufficient bone in the left zygomatic region. Therefore, it was decided to design patient-specific implants (PSI) according to the availability of bone.

**Fig. 4:**
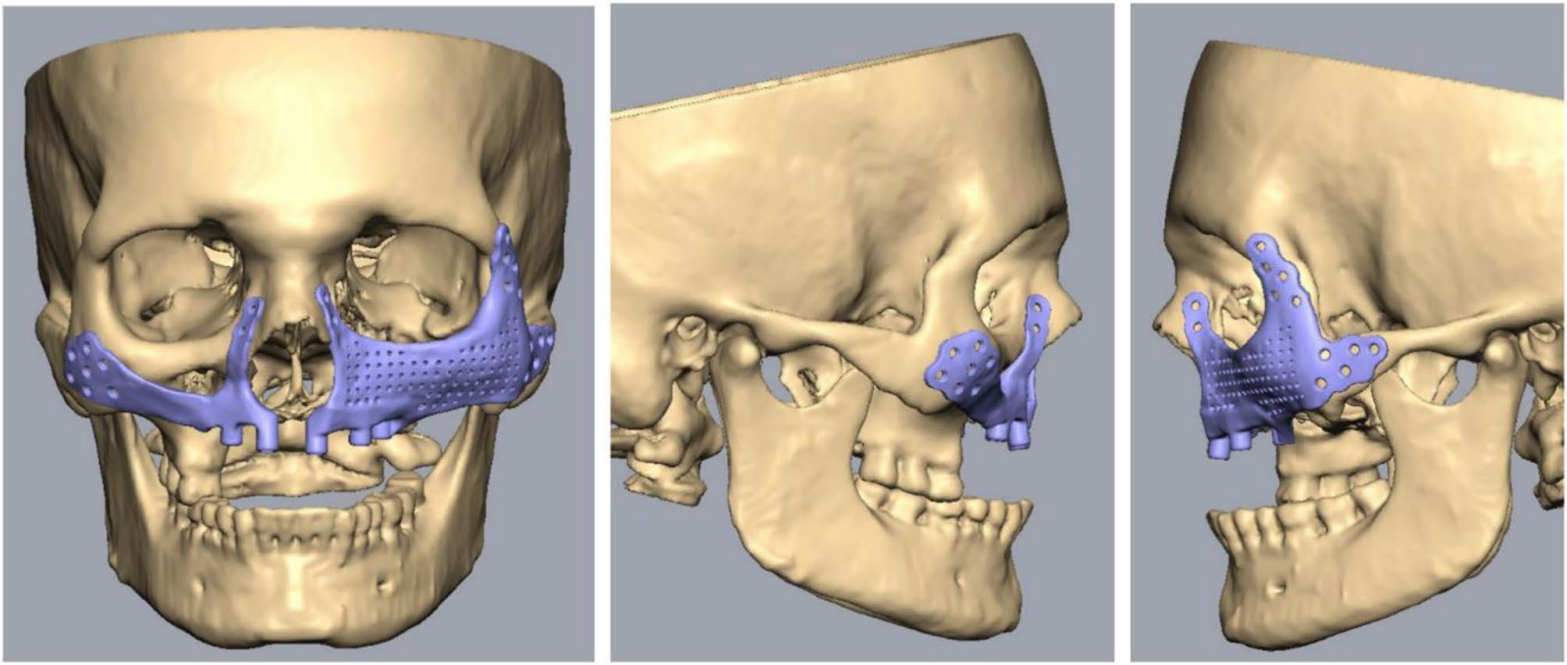
Designing of patient-specific implants

**Fig. 5:**
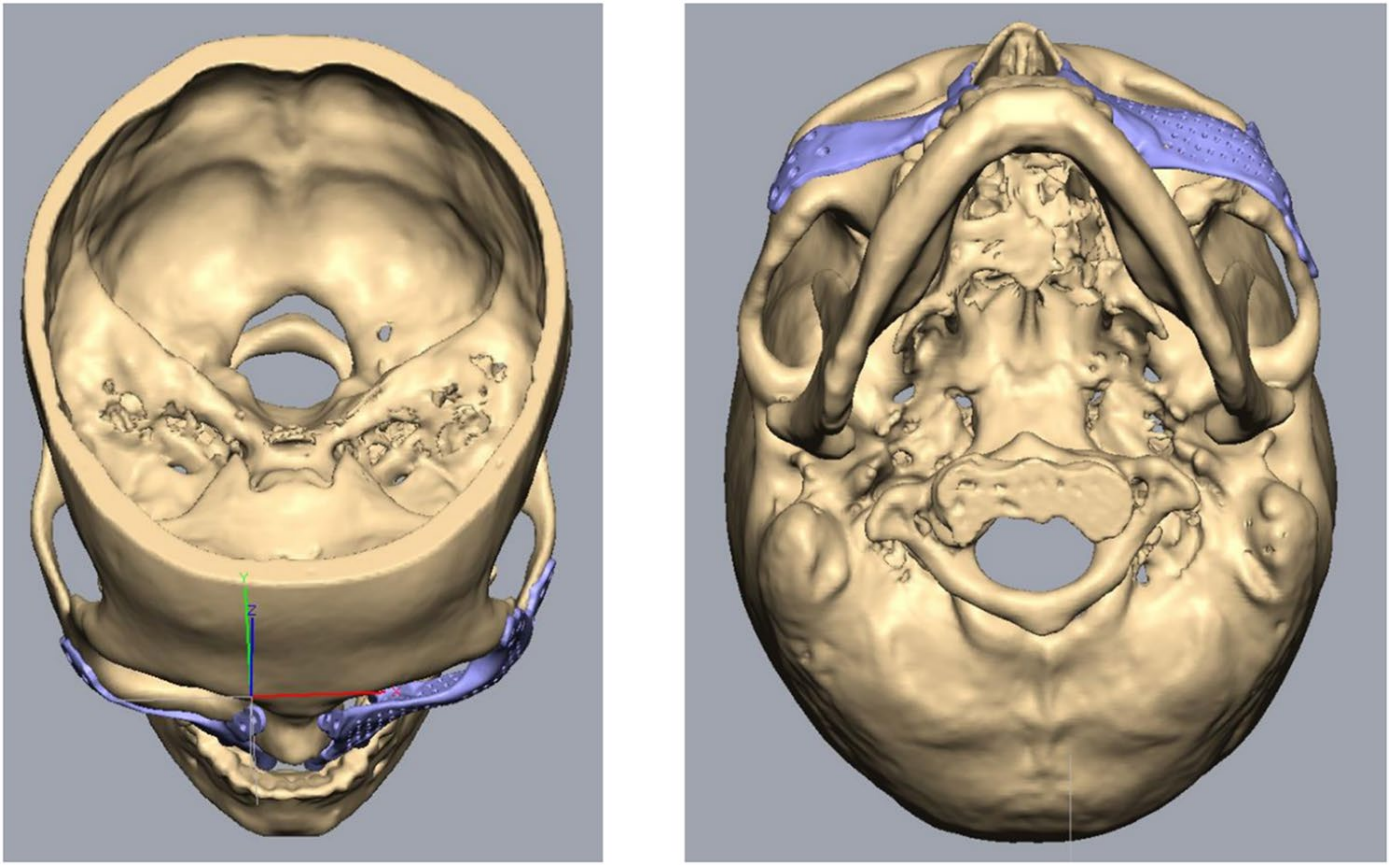
Top and bottom view

**Fig. 6:**
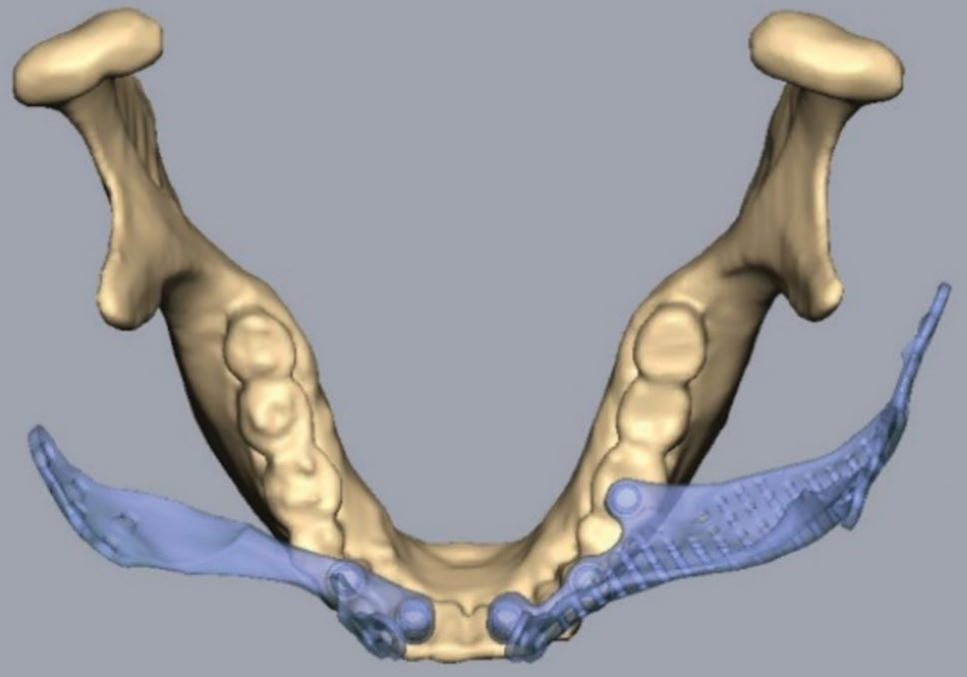
Occlusal comparison of PSI with the opposing arch

**Fig. 7:**
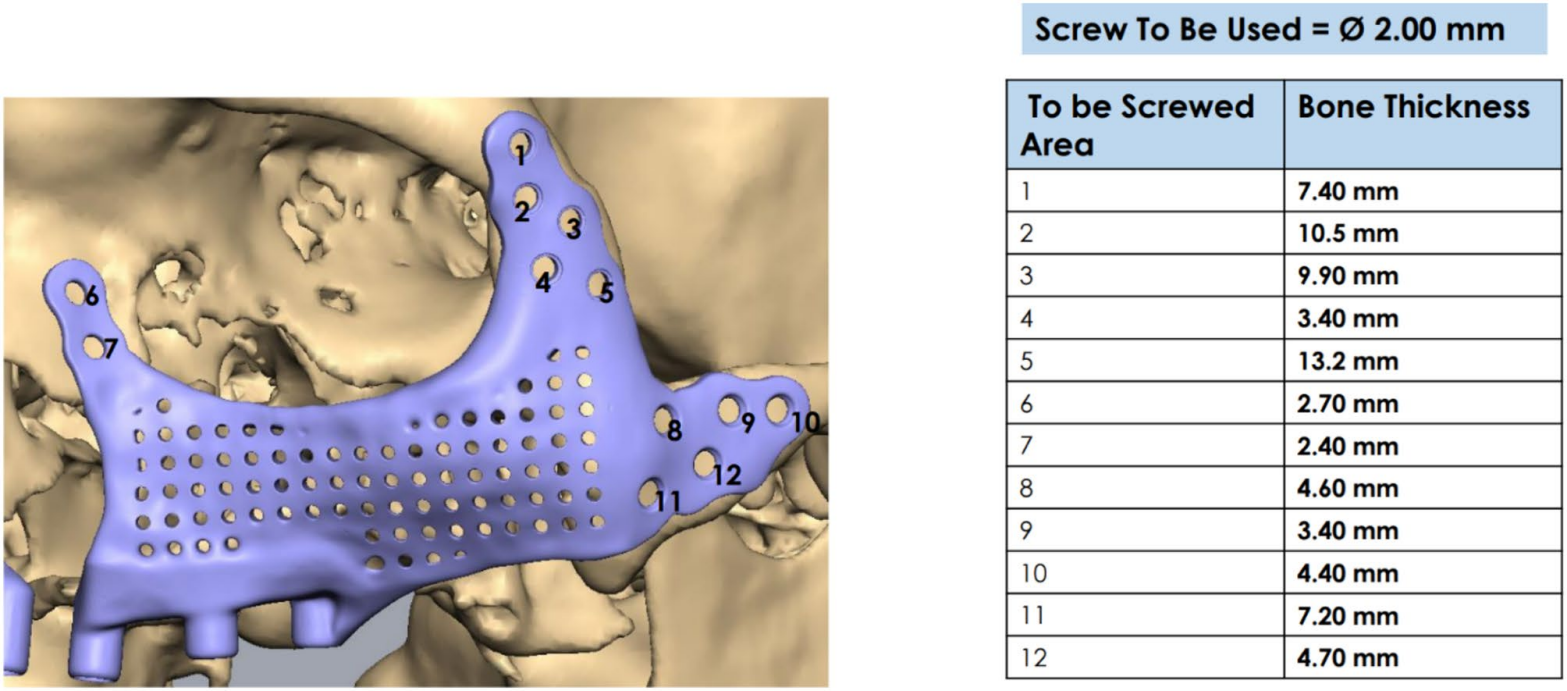
Specification of PSI on the left side

**Fig. 8:**
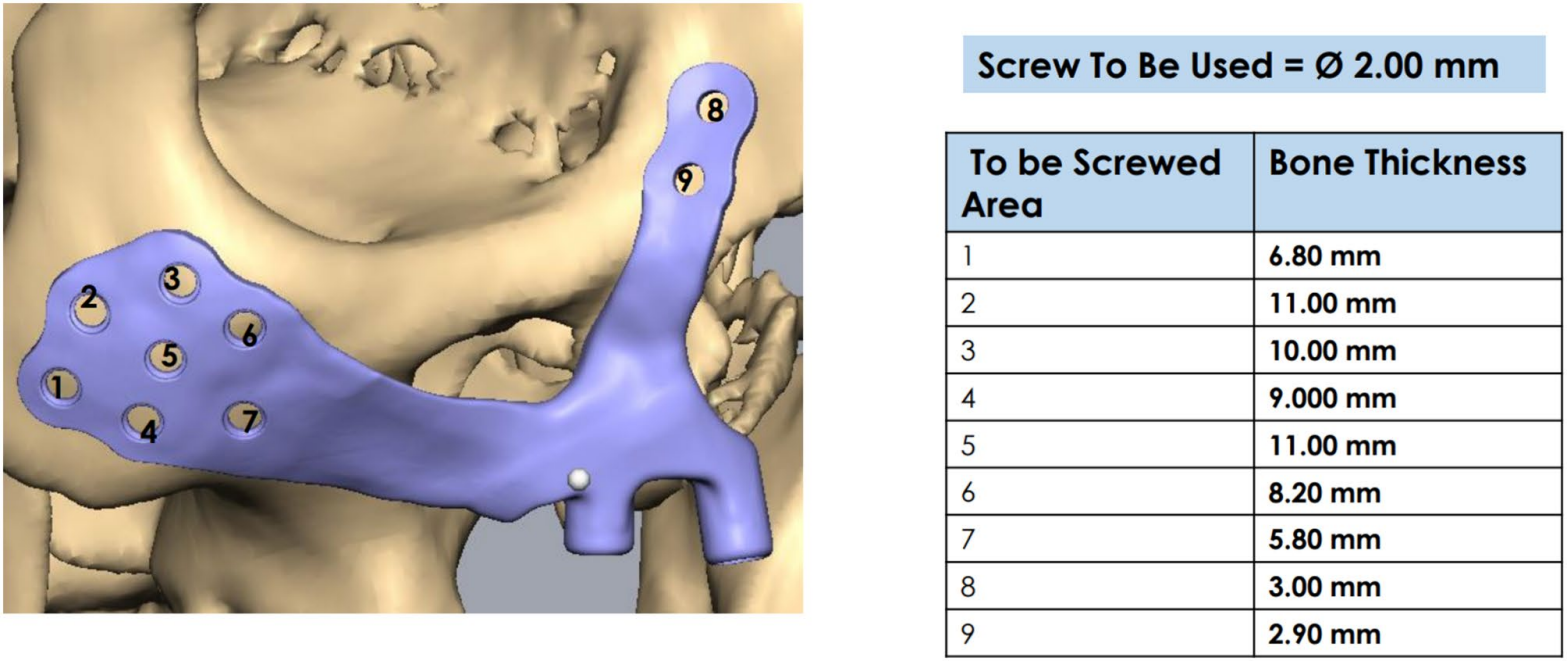
Specification of PSI on the right side

**Fig. 9:**
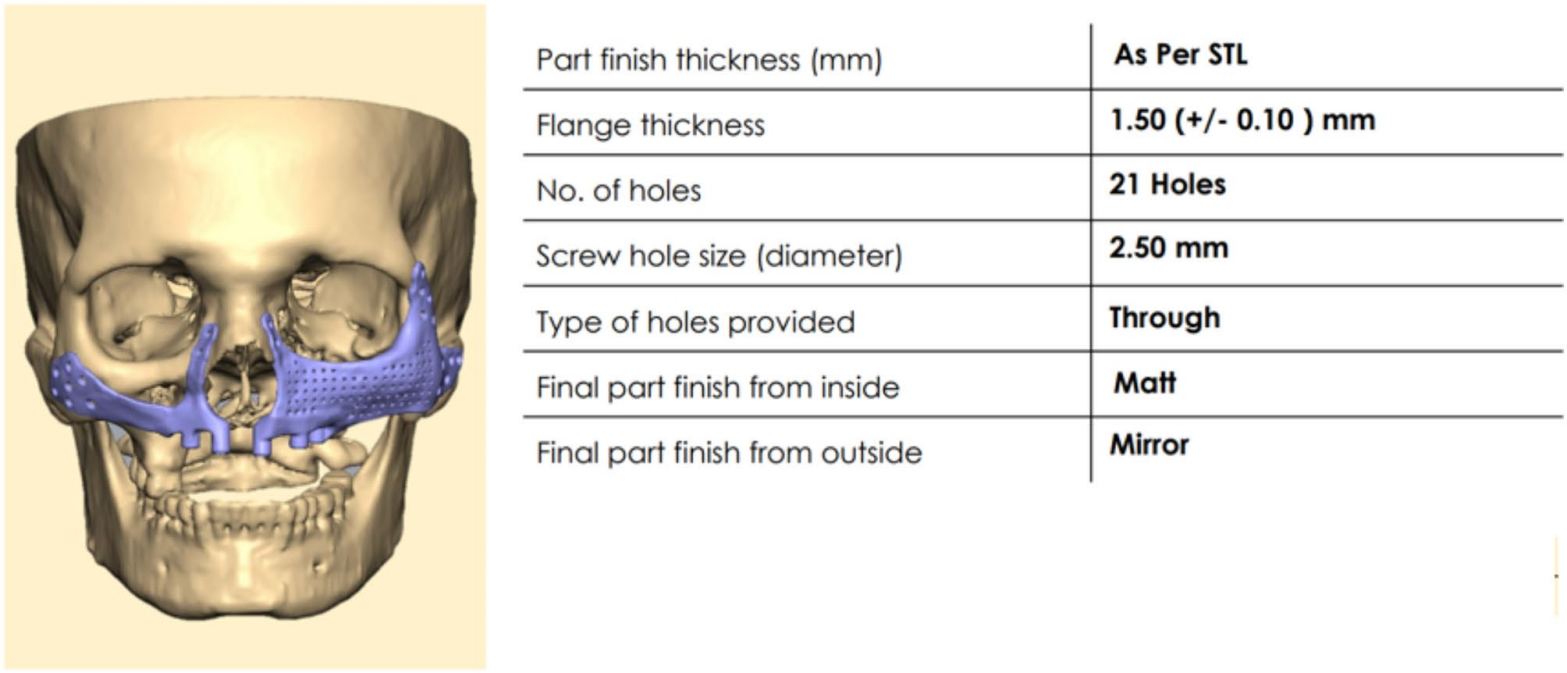
Planning and designing of PSI

For PSI’s surgical and prosthetic components, virtual surgical planning and design were considered to maximize effectiveness and sustainability (Figs. 4, 5, 6, 7, 8 and 9).

**Fig. 10:**
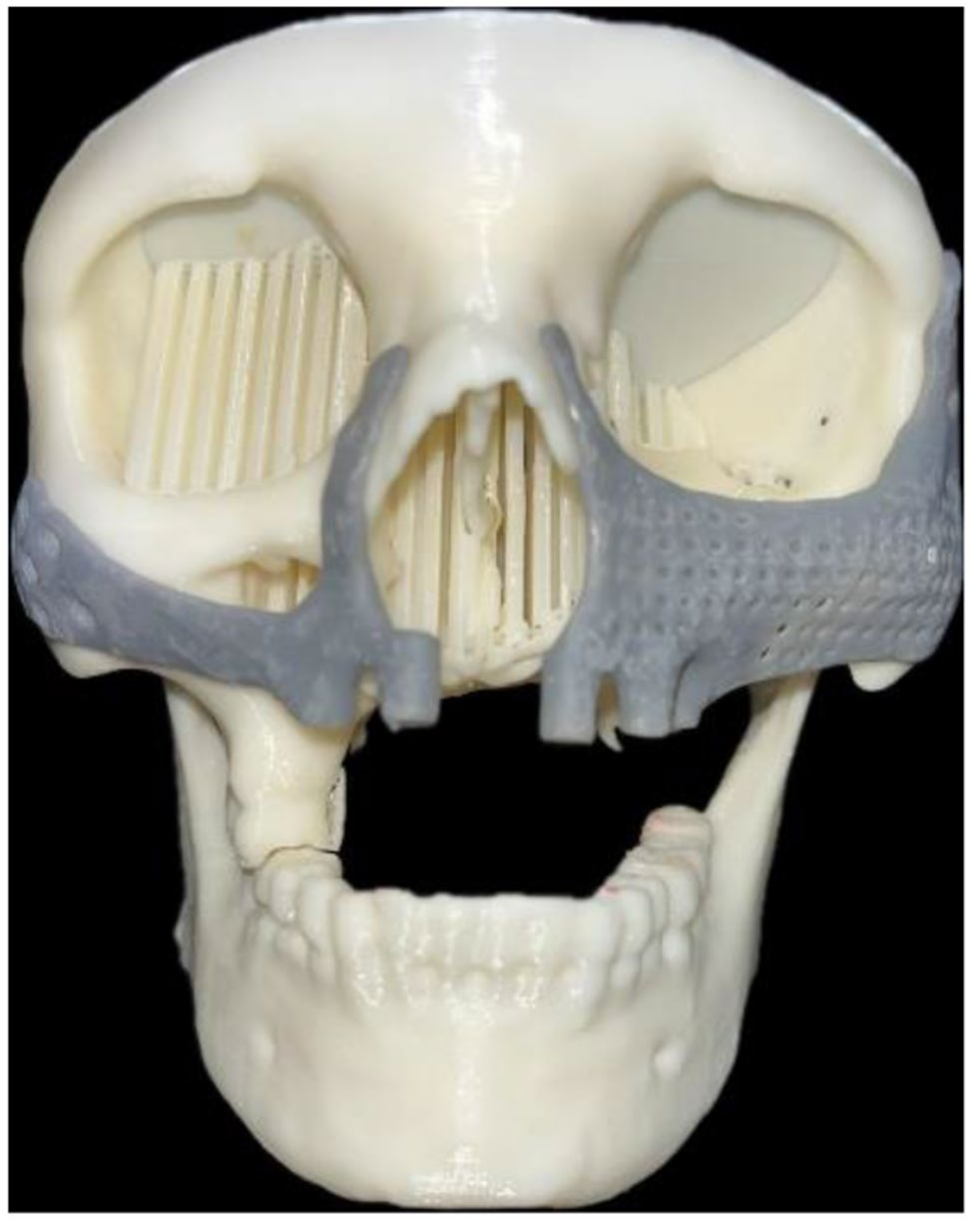
3D printed model

After the treatment plan and final decision were made, the 3D projection of the maxilla was saved as a standard tessellation language (STL) file to the implant’s computer-aided design (CAD). A 3D-printed model with PEEK material was fabricated for evaluation and study (Fig. 10).

After the doctors approved the final design, the implant was 3D printed using grade 5 titanium alloy powder (Ti6Al4V) to produce the actual titanium PSI. This technique, called *selective laser sintering*, produces incredibly accurate results by selectively fusing titanium powder with a laser beam.

**Fig. 11:**
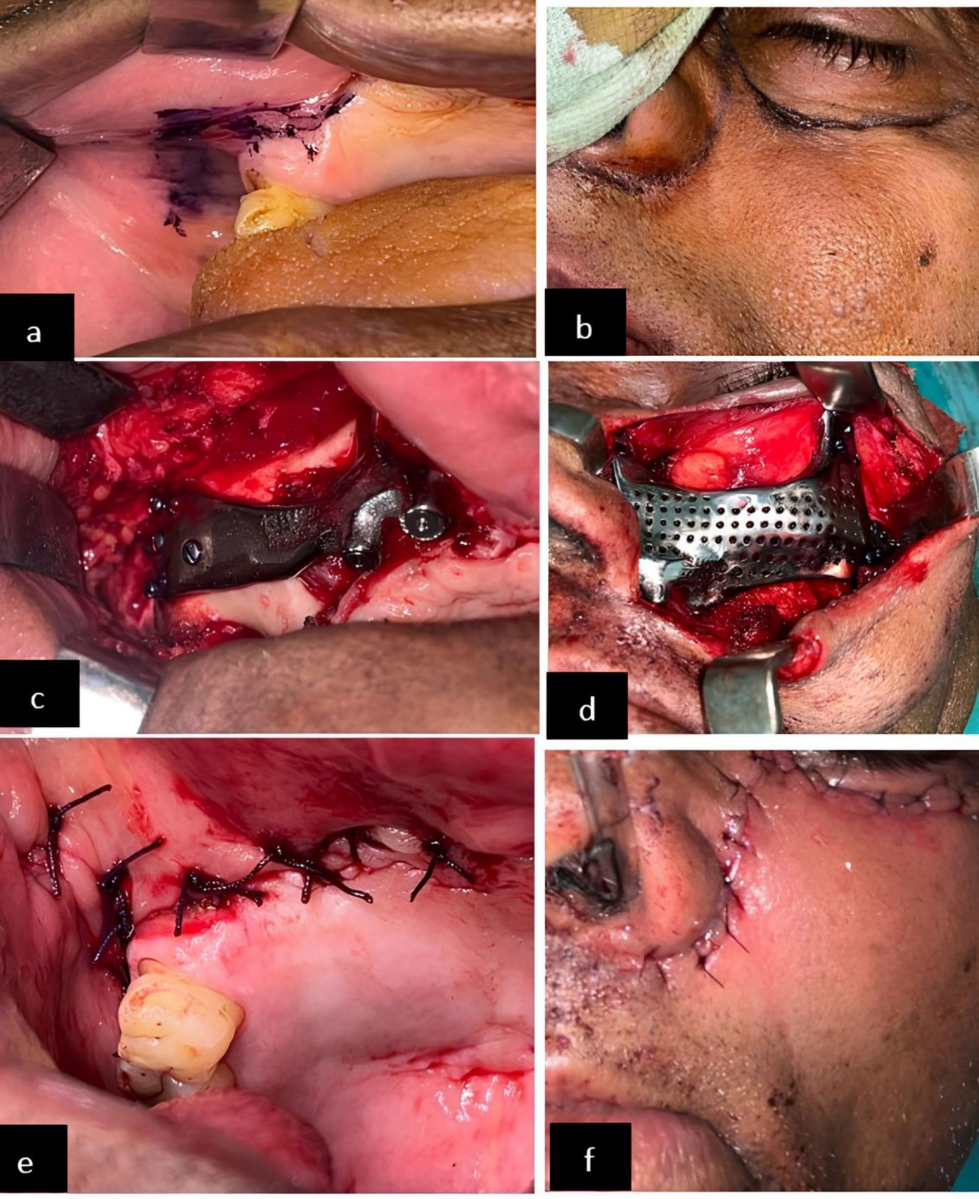
**a**: incision marking on the right side, **b**: Weber Ferguson incision marking on the left side, **c**: implant placement on the right side, **d**: implant placement on the left side, **e**: closure with absorbable suture on the right side, **f**: closure with absorbable suture on the left side

General anesthesia was administered for the patient’s operation. After locating the parotid duct, a 15-mm vestibular incision was made on the right side, directly across the zygoma body and away from the fibrosed and retracted palatal mucosa. An extraoral Weber-Ferguson incision was made on the left side. Sub-periosteal undermining was done up to the frontozygomatic suture and the root of the zygomatic arch across the zygoma body. Bilateral implants were fitted in a pocket formed by sub-periosteal undermining over the zygoma body. Screws were used to secure the titanium bar and framework, which connected the implant bar’s two sides (Fig. 11).

Following a re-evaluation of the implants’ compatibility with the zygoma body, self-drilling titanium screws were used to secure the implants on both sides. Virtual surgical planning was used to predetermine the screw size. Using a trocar, screws at the lateral orbital rim were fixed. Nine to twelve screws were utilized on each side to ensure the implant was as stable as possible. Mucograft was used to promote soft tissue recovery towards the bottom of the implant bar. The implant surface was covered with a buccal pad of fat to allow for proper soft tissue recovery. Resorbable sutures were used to close the wound while leaving a self-cleaning region unharmed. Forty-eight hours following surgery, a postoperative CT scan was acquired to assess how well the implant had adapted to the zygoma body.

**Fig. 12:**
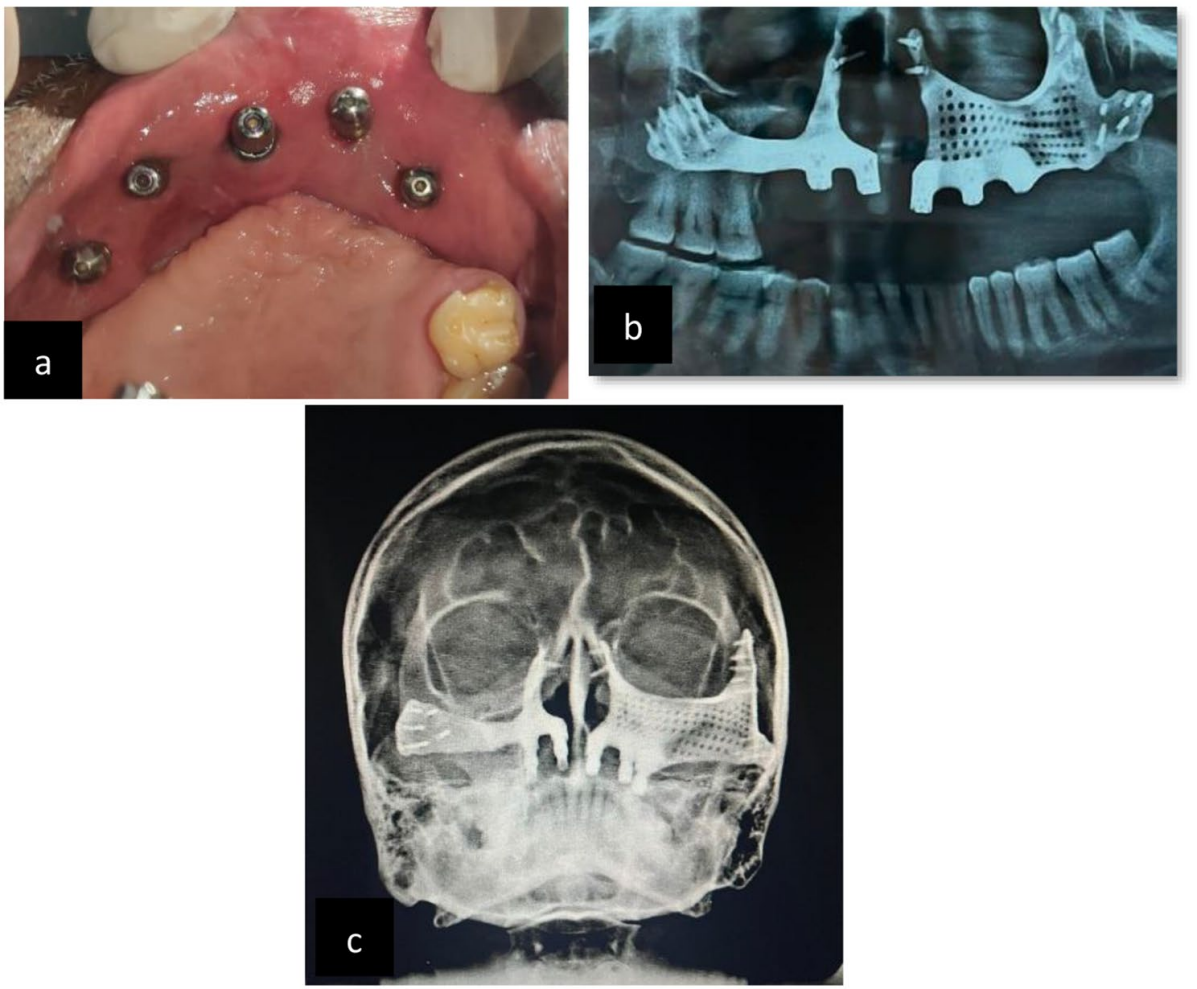
**a**: post-operative intraoral view, **b**: post-operative OPG, **c**: Waters view

**Fig. 13:**
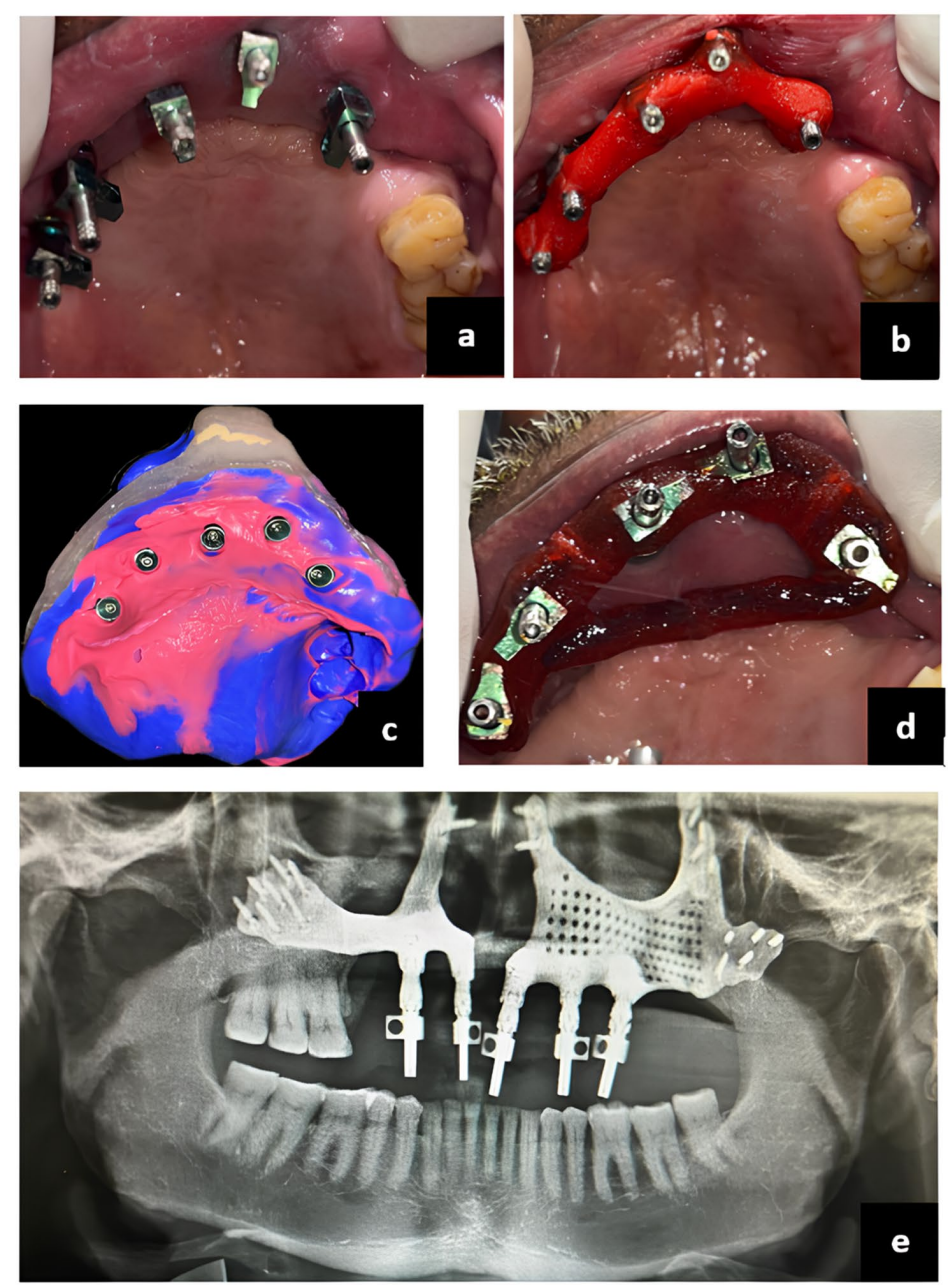
**a**: Open tray impression copings attached to the multiunit abutments, **b**: Pattern resin adapted to the floss tied to the impression copings, **c**: Open tray impression with Light body and putty consistency of polyvinyl siloxane (PVS) impression material. **d**: Jig attached to the multiunits, **e**: OPG showing Jig verification

**Fig. 14:**
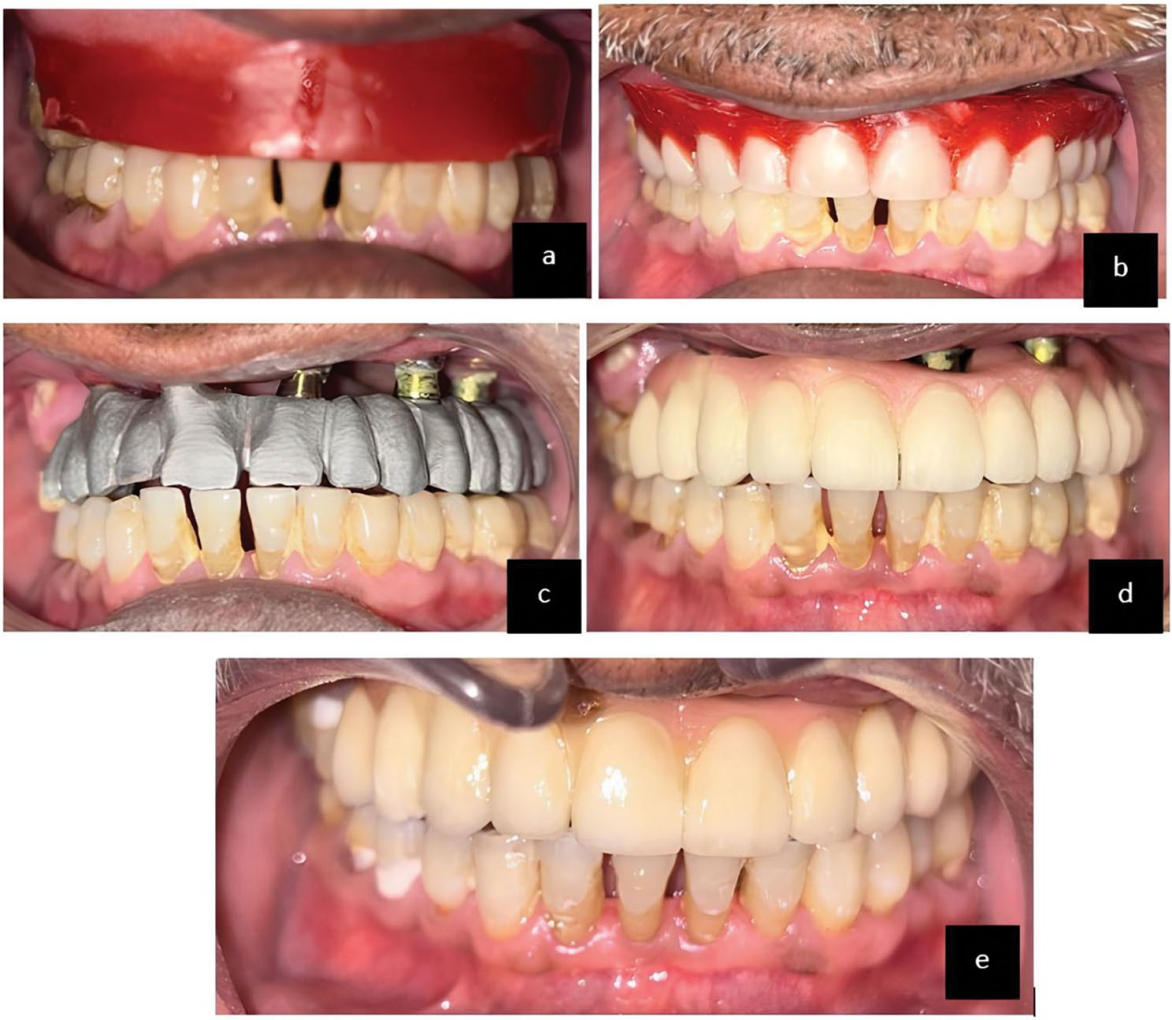
**a**: Jaw relation, **b**: teeth arrangement, **c**: metal try, **d**: Bisque trial, **e**: Final Prosthesis

Technical details of prosthesis planning include an FP-3 type design. A delayed loading was carried out after 1 month. In the prosthetic phase, the patient was brought back one month later for the definitive prosthesis. After recording an open-tray impression, the metal framework was fabricated in the laboratory. Following the jaw relation procedure, a try-in was performed, and a hybrid prosthesis was loaded to close the defect. Successive follow-up visits were scheduled for the patient. After 15, 30, 45, and 90 days and every month thereafter, the patient was examined to assess possible complications. Patients had follow-up periods ranging from six months to a year (Figs. 12, 13, and 14).

## Discussion

Patient-specific implants (PSIs) represent a significant advancement in oral surgery, enabling simultaneous dental and maxillofacial rehabilitation to achieve immediate aesthetic and functional outcomes [1, 6]. Leveraging 3D printing and CAD-CAM technology, the reconstruction of complex anatomical structures, including 3D facial forms, has become more accessible [7]. Additive manufacturing techniques facilitate the creation of custom-designed devices tailored to specific reconstructive requirements [1, 3], overcoming limitations associated with autologous bone grafts [2, 8]. Following surgical placement of the PSI, an FP-3 type definitive prosthesis was positioned over multi-unit abutments.

In the presented case report, due to mucormycosis, the patient had undergone maxillectomy. Due to this resection, no retentive features remained in the patient’s maxilla. Decreased vestibular depth and a shallow palatal vault might lead to a loss of retention in the maxillary arch. Therefore, a removable prosthesis was not an option for this patient. Following the complete diagnosis and treatment planning, inadequate bone was noted in the maxilla and zygomatic region, so it was decided to use customized implants for the patient.

While PSI offers precision, surgical experience is essential, and bone contouring may be necessary for proper fit and placement due to limited intraoral access. In the reported case, a combined intraoral and extraoral approach was preferred, though this might cause aesthetic concerns and scar formation. The challenge of achieving good primary closure of the PSI in cases with limited soft tissue was addressed by undermining the soft tissue. Post-COVID mucormycosis often results in significant anatomical defects, presenting challenges for maxillofacial surgeons.

For sub-periosteal implants, titanium is preferred over chrome-cobalt alloys because it improves tissue compatibility and reduces implant failure, exposure, or rejection [3, 7]. While digitally produced implants were placed using vestibular incisions directly over the zygomatic body, conventional sub-periosteal implants were implanted using mid-crestal incisions, which provided enough exposure and soft tissue for closure without wound dehiscence [5]. Using a trans-buccal trocar and self-drilling screws, fixation was accomplished once the implant was properly adapted. This improved fixation by making it easier to position screws perpendicular to the plate [8]. Zygomatic implants represent a significant advancement in oral implantology, particularly for patients with severe maxillary atrophy, where traditional implant placement may be challenging [12–16]. A study was conducted to evaluate the clinical outcome of zygomaticus implants, demonstrating favorable results with a 6–8 month follow-up period. These findings provide valuable insights into the efficacy and stability of zygomatic implants, supporting their use as a viable treatment option in complex maxillofacial cases [17].

The future of reconstruction, known as PSI, will enable simultaneous dental and maxillofacial rehabilitation to immediately improve both functional and aesthetic features. The complicated structure of the craniofacial region, as well as the functional and aesthetic effects on patients, make reconstruction of these anomalies extremely difficult. Patient-specific implants (PSIs) utilizing virtual surgical planning based on a computer-aided design (CAD)/computer-aided manufacturing (CAM) platform are promising treatment options thanks to emerging technologies. Patients undergoing reconstruction or with maxillofacial deformities can benefit from PSIs. Preformed plates made from PSIs are also utilized in virtually planned orthognathic operations. As part of alloplastic joint replacement, customized temporomandibular joint (TMJ) prostheses are frequently used in degenerative/debilitating joint disorders [18].

In the case report previously mentioned, an intraoral incision was made to the right side of the zygomatic buttress region, and an incision was made on the left side to include the implant through a Weber-Ferguson incision. Outstanding surgical access is provided by this kind of incision. As the preferred incision for exposing the orbit or maxilla, the Weber-Ferguson technique has received a lot of attention. The infraorbital neurovascular bundle, which is often transected and reapproximated after the treatment, may restrict access to the most lateral aspect of the maxilla and infratemporal fossa [19]. The Weber-Ferguson incision and the midface degloving incision are two of the frequently used routes to the maxillary region. There is debate over who initially characterized the incision. Several researchers believe that Gensoul was the first to describe it in 1893 and that Weber & Fergusson later popularized the concept. Some contend that Weber originally discussed it in German writing, and Fergusson later made modifications in English literature. Furthermore, many think that Weber and the method were first described by Sir William Fergusson [20].

In recent times, the idea of a “one-size-fits-all” strategy has given way to a personalized one. Technological advancements such as computer-aided design and computer-aided manufacturing (CAD/CAM) have made it possible to customize a surgical procedure to a patient’s specific demands, even in cases involving complicated facial skeletons, which have improved surgical outcomes [20]. In our instance, PSI has been produced with the use of selective laser sintering. A collaborative method of implant design is being used, involving real-time communication between the surgeon and implant designer to generate precisely tailored PSI to match the bone deficiency. This approach is made possible by advancements in processing power and 3D modeling software.

Thus, the approach has revolutionized the therapeutic strategy for correcting residual deformities/defects in the maxillofacial region through the creation of patient-specific implants, or PSIs. PSIs are being employed in prefabricated fixation implants for orthognathic surgery, as well as in the form of temporomandibular joint (TMJ) entire replacement and reconstruction of the orbital, cranial, maxillectomy, and mandibulectomy abnormalities, post-operative radiation therapy leading to osteoradionecrosis causing large bony defects. A large body of research suggests that these implants are being used more frequently than alternative forms of treatment. Sometimes oncologic resections of mild to moderate size result in noticeable abnormalities in the craniofacial area. Reconstruction in these circumstances is often difficult, particularly when using autogenous options such as vascularized bone transplants. Even though they have been the gold standard for reconstruction, there is always a risk of tissue necrosis following high-dose anticancer chemotherapy and radiation, as well as a limit on the amount of bone that may be used. Such post-cancerous deformities require large-scale reconstruction, including bony regions, which can be handled using an implant strategy tailored to the patient [18].

The patient’s edentulous facial look was corrected without causing noticeable scars by the use of prosthetics providing enough lip support, minor stab incisions for trocar insertion, and intraoral vestibular incisions. Patients can acquire functional prostheses in a single surgical step because of the digitally developed design. In the case mentioned above, the prosthesis is loaded on bilateral plates of implants, which are splinted with a metal bar. Cross-arch stabilization is achieved, and stress on the implants is equally distributed. Using the osseofixation principle, the prosthesis connection system that is integrated with the implant enables quick insertion over the bar that is attached to the implant. Using proven methods, prosthodontic rehabilitation with PSI appears to have success rates similar to those of traditional quad zygoma implants [4].

In a shorter amount of time and with less surgical morbidity, patient-specific implants provide the best stability and functional rehabilitation for patients who have undergone mucor maxillectomy. To more accurately evaluate their advantages, though, longer follow-ups and larger sample numbers are required in future research.

## Conclusion

The resulting PSIs enable the prosthesis to be loaded non-functionally right away, providing adequate occlusal and cosmetic rehabilitation. The following case report demonstrates the effective use of PSIs in maxillofacial surgery to restore a sizable defect caused by a hemi-maxillectomy. The combination of CAD-CAM and 3D printing technologies has significantly enhanced the ability to produce precise and personalized PSIs.

## Electronic Supplementary Material

Below is the link to the electronic supplementary material.

Supplementary Material 1

## Data Availability

The data will be available on figshare under the following DOI 10.6084/m9.figshare.25715697.

## Abbreviations

PSI: Patient-specific implants
CT: Computed tomography
CAD/CAM: Computer-aided designing and computer aided milling
