# Supplementary material for: Outstretching challenges for rehabilitation of a mucormycotic case-a digitally designed patient-specific implant approach in the recent era
Source: BMC Oral Health. 2024 Nov 15;24:1383. doi: 10.1186/s12903-024-05099-4 (PMC11566662; doi:10.1186/s12903-024-05099-4)
Supplement: Supplementary file 1 — Supplementary Material 1 [file 12903_2024_5099_MOESM1_ESM.pdf]

LUCID

Original View

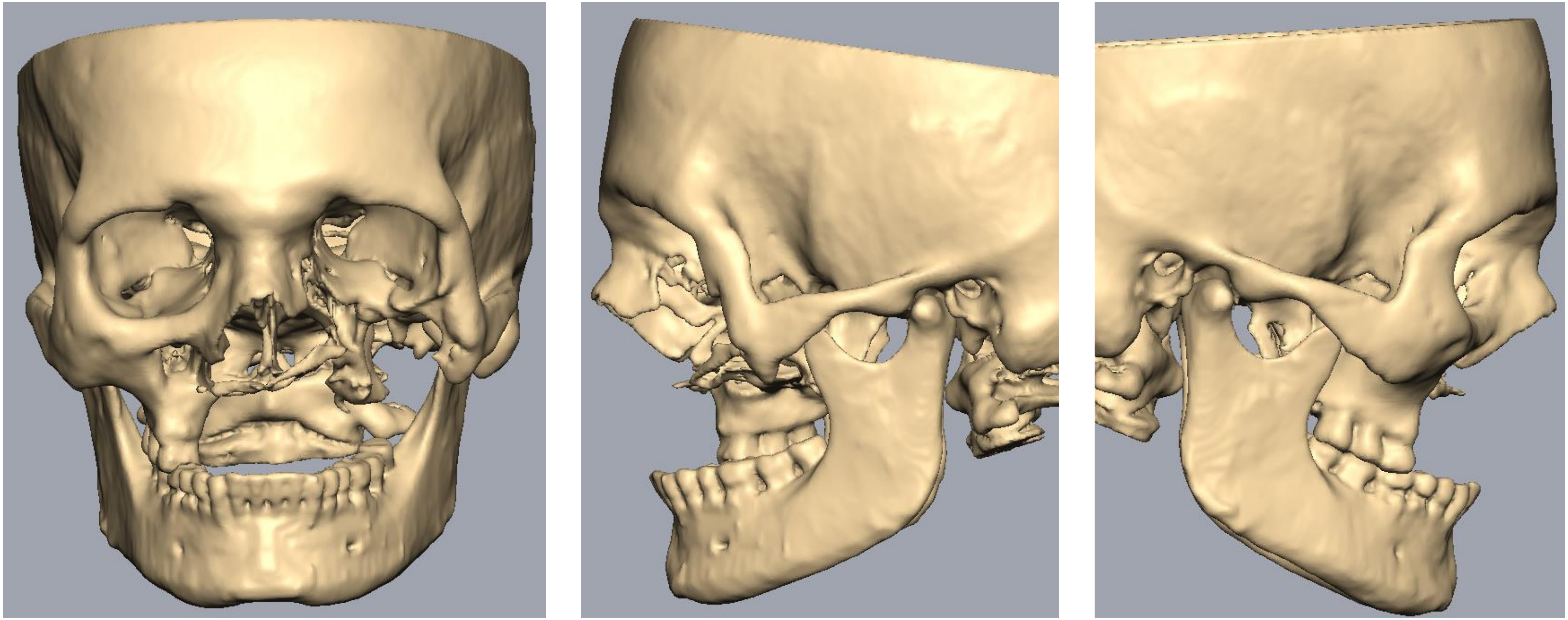

Implant View

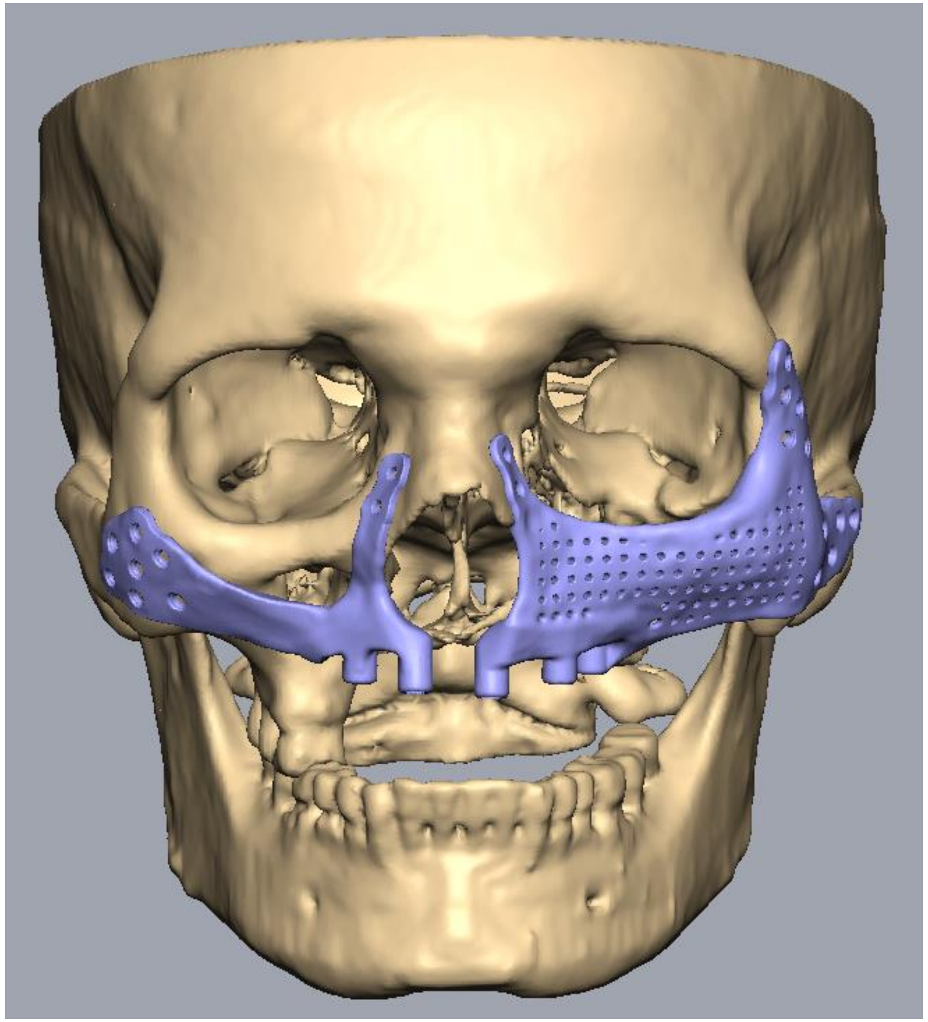

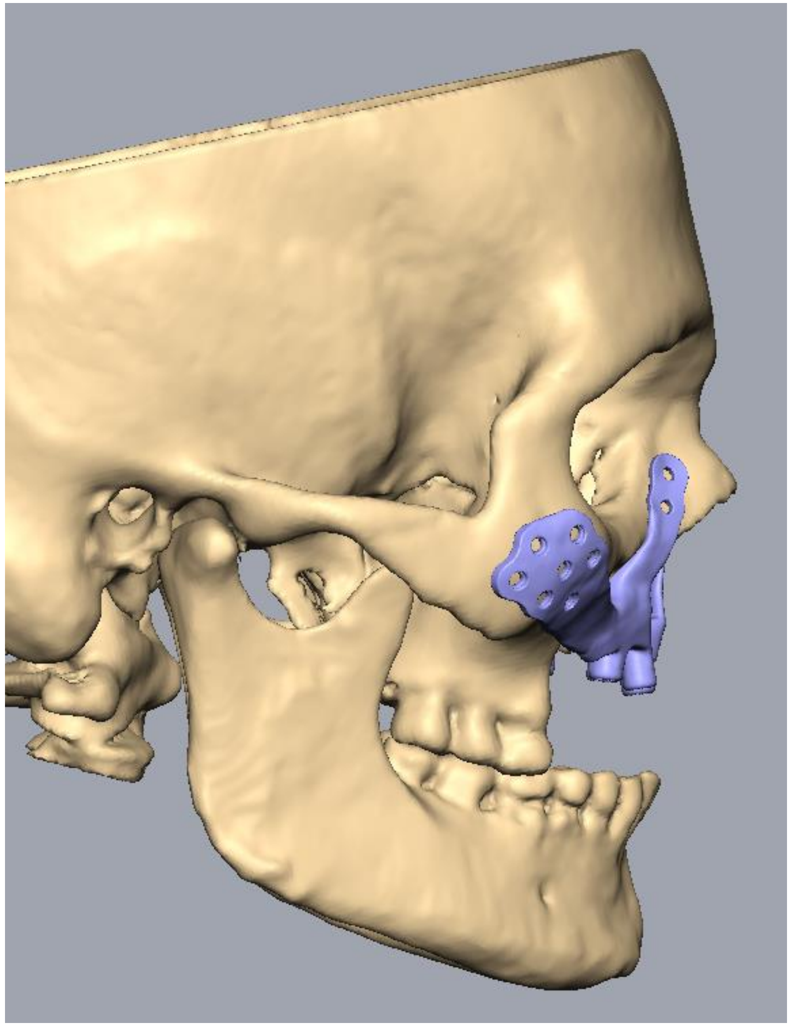

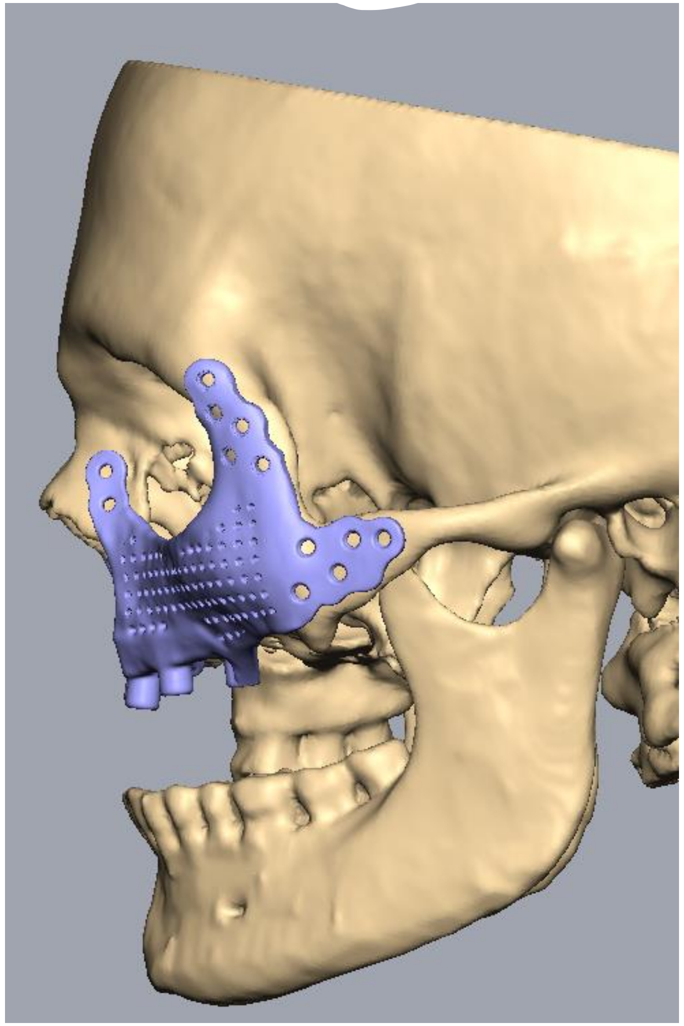

LUCID

Top & Bottom View

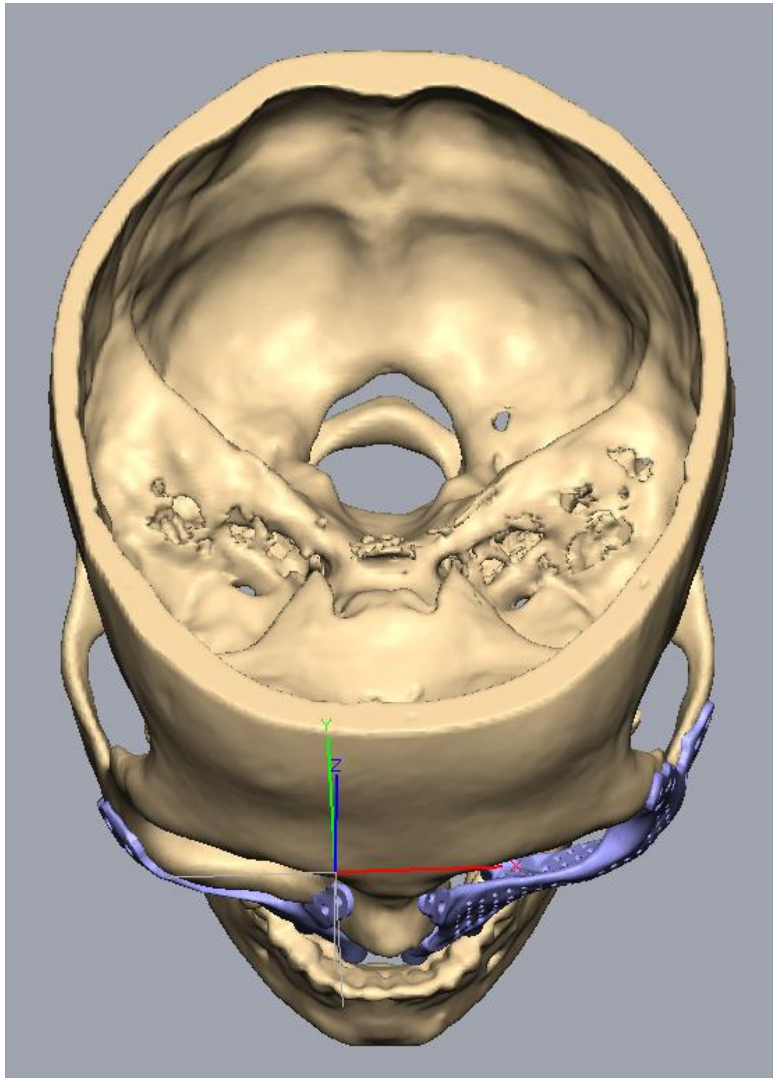

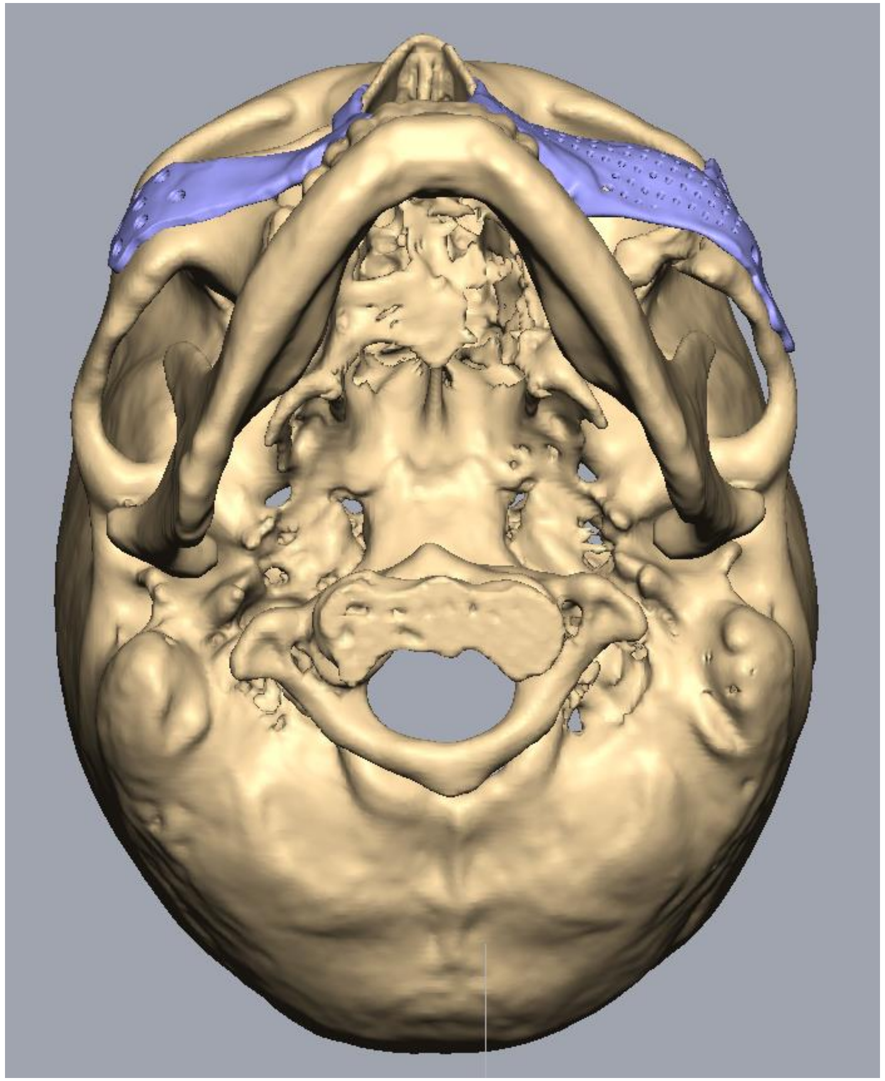

Comparative View

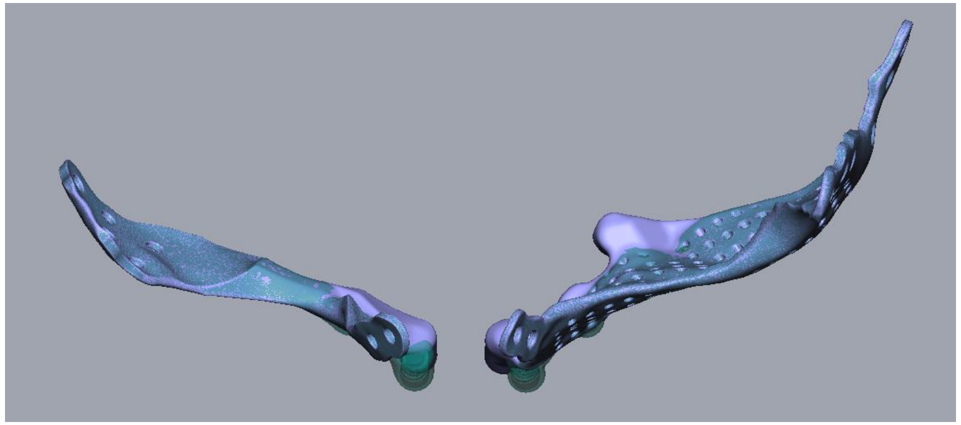

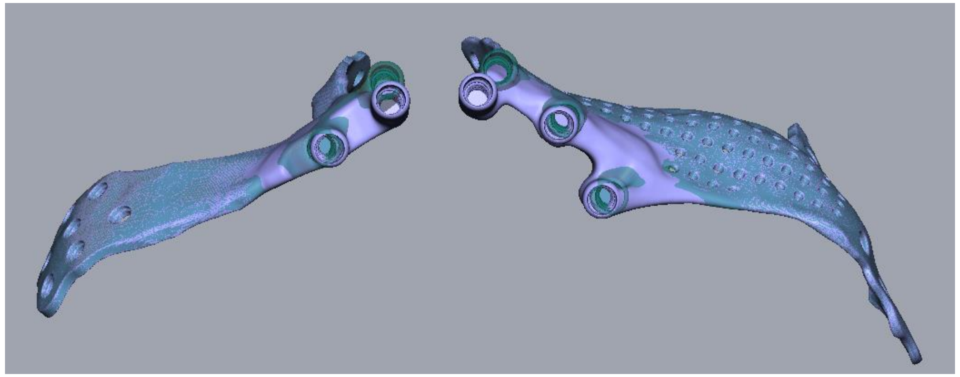

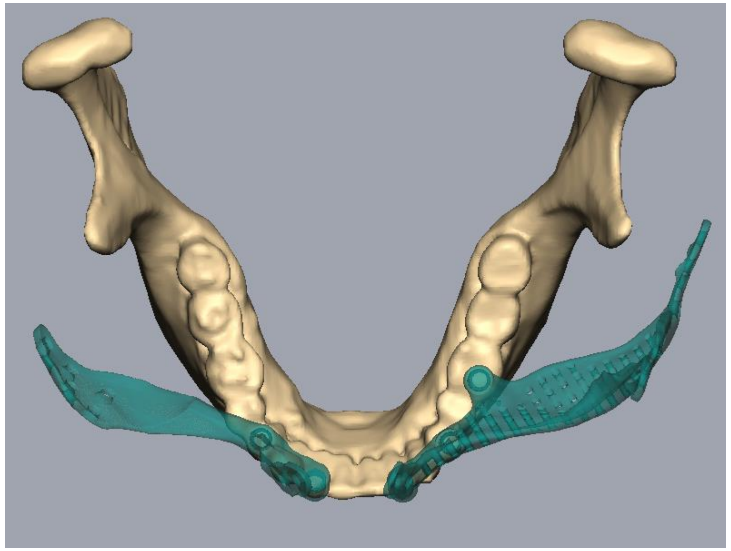

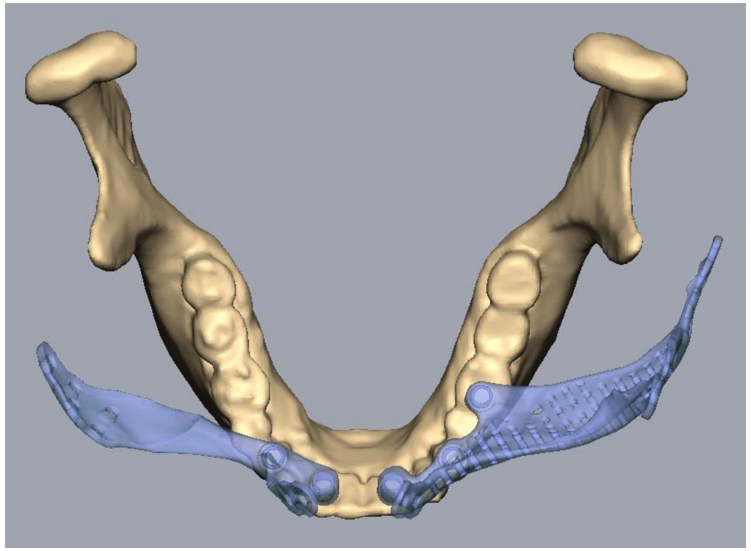

LUCID

Detailed View

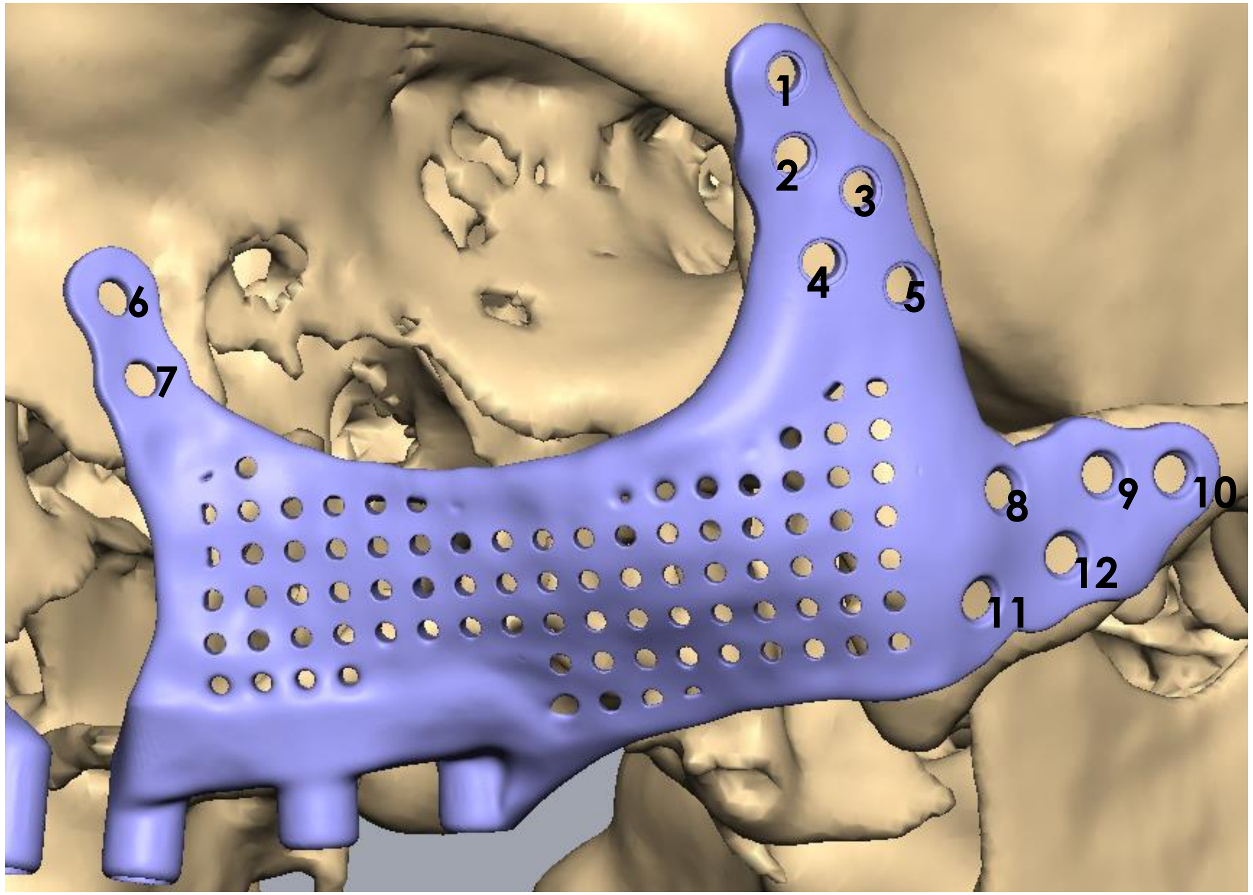

Screw To Be Used = Ø 2.00 mm

To be Screwed Area	Bone Thickness
1	7.40 mm
2	10.5 mm
3	9.90 mm
4	3.40 mm
5	13.2 mm
6	2.70 mm
7	2.40 mm
8	4.60 mm
9	3.40 mm
10	4.40 mm
11	7.20 mm
12	4.70 mm

Detailed View

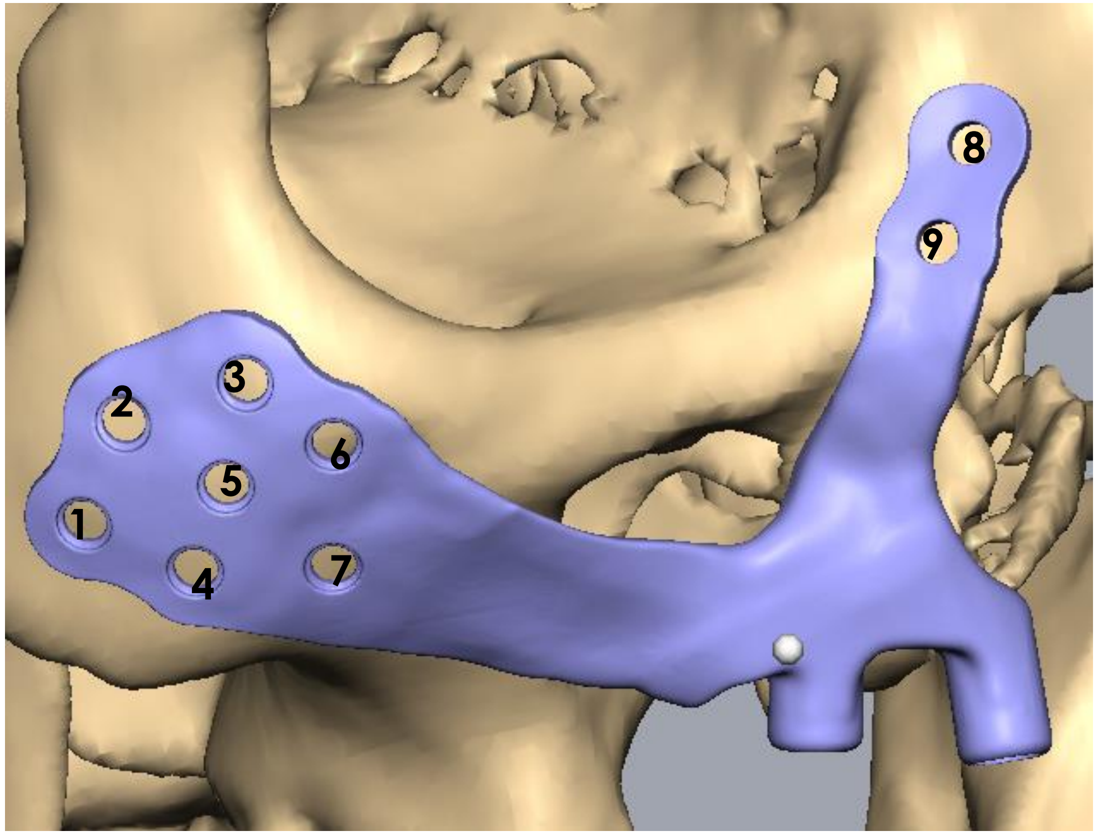

Screw To Be Used = Ø 2.00 mm

To be Screwed Area	Bone Thickness
1	6.80 mm
2	11.00 mm
3	10.00 mm
4	9.000 mm
5	11.00 mm
6	8.20 mm
7	5.80 mm
8	3.00 mm
9	2.90 mm

LUCID

Technical Sheet

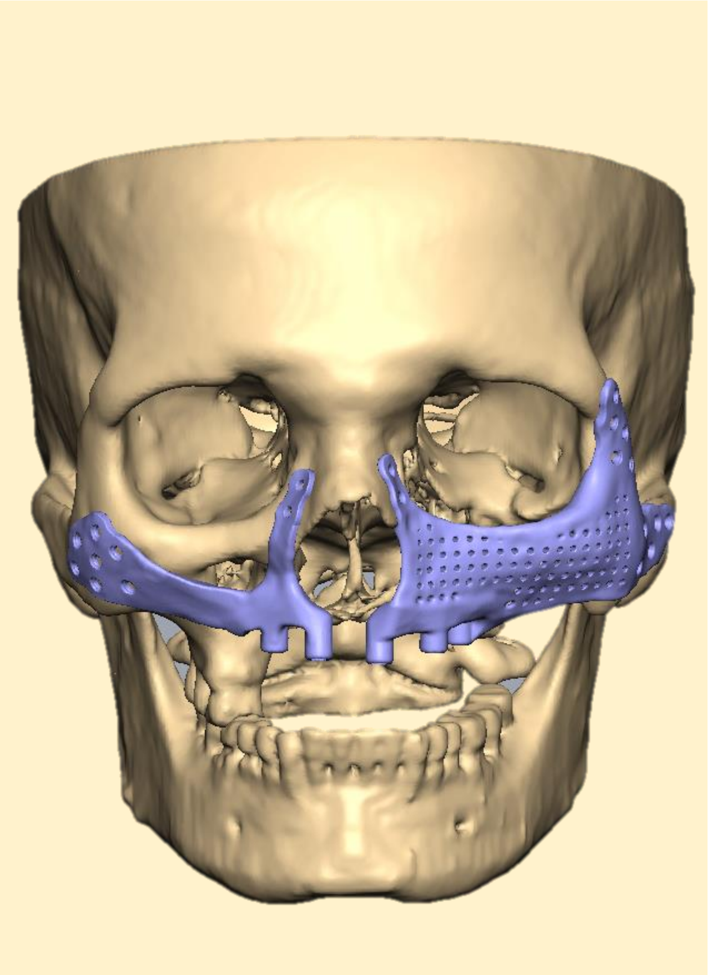

Part finish thickness (mm)	**As Per STL**
Flange thickness	**1.50 (+/- 0.10) mm**
No. of holes	**21 Holes**
Screw hole size (diameter)	**2.50 mm**
Type of holes provided	**Through**
Final part finish from inside	**Matt**
Final part finish from outside	**Mirror**
